# Peer Review of “The Influence of SARS-CoV-2 Variants on National Case-Fatality Rates: Correlation and Validation Study”

**DOI:** 10.2196/38522

**Published:** 2022-05-24

**Authors:** Ramesh Poluru

**Affiliations:** 1 The INCLEN Trust International New Delhi India

**Keywords:** SARS-CoV-2, COVID-19, variants of concern, case-fatality rates, virulence, vaccine effectiveness, correlation study

*This is a peer-review report submitted for the paper “The Influence of SARS-CoV-2 Variants on National Case-Fatality Rates: Correlation and Validation Study.”*

## Round 1 Review

I would like to appreciate the author for this study [1] addressing the influence of SARS-CoV-2 variants on national case-fatality rates. The manuscript is concise and well written, and is recommended for possible consideration in its current form. Before publishing the manuscript, I suggest the author presents an Appendix with (a) data with absolute numbers, (b) illustration for smoothed values of the proxy case-fatality rate for at least one country (Figures 8-11), and (c) alternatively discussion on the analytical framework in detail in the Method of Analysis section.

In conclusion, the subject addressed in this manuscript is worth investigation, and the manuscript is recommended for possible consideration after addressing the above minor concerns.
